# Calpain inhibition by calpeptin modulates adipocyte lipid metabolism and secretome-mediated inflammatory crosstalk with hepatocytes

**DOI:** 10.1007/s00011-026-02280-z

**Published:** 2026-06-04

**Authors:** Johanna Matilainen, Sanni Foster, Viivi Berg, Anne-Mari Mustonen, Sanna Oikari, Kirsi Rilla, Reijo Käkelä, Sanna P. Sihvo, Petteri Nieminen

**Affiliations:** 1 Institute of Biomedicine, School of Medicine, Faculty of Health Sciences, University of Eastern Finland, Kuopio, Finland; 2 Obesity Research Unit, Research Program for Clinical and Molecular Metabolism, Faculty of Medicine, University of Helsinki, Helsinki, Finland; 3 Department of Environmental and Biological Sciences, Faculty of Science, Forestry and Technology, University of Eastern Finland, Joensuu, Finland; 4 Department of Technical Physics, Faculty of Science, Forestry and Technology, University of Eastern Finland, Kuopio, Finland; 5 School of Pharmacy, Faculty of Health Sciences, University of Eastern Finland, Kuopio, Finland; 6 Department of Molecular and Integrative Biosciences, Faculty of Biological and Environmental Sciences, University of Helsinki, Helsinki, Finland; 7 Helsinki University Lipidomics Unit (HiLIPID), Helsinki Institute of Life Science (HiLIFE) and Biocenter Finland, Helsinki, Finland

**Keywords:** Adipocyte, Calpeptin, Extracellular vesicle, Fatty acid, Inflammation, Palmitic acid

## Abstract

**Objective:**

Calpeptin, a calpain inhibitor with potential for treating inflammatory diseases, reduces extracellular vesicle (EV) secretion. While adipose tissue is a recognized target of calpeptin, its effects on lipid metabolism remain unknown. We investigated calpeptin’s impact on fatty acid (FA) profiles and metabolic pathways in human Simpson-Golabi-Behmel Syndrome adipocytes and their EVs.

**Methods:**

Adipocytes were treated with 25 or 50 µM calpeptin, and EVs were isolated from conditioned media (CM) by ultracentrifugation. Immortalized human hepatocytes (IHHs) were pre-treated with 0 or 400 µM palmitic acid (PA) and subsequently exposed to CM from calpeptin-treated adipocytes. Total lipid FA composition was determined by gas chromatography–mass spectrometry, and gene expression with RNA-sequencing and qPCR, followed by univariate and multivariate statistics and pathway analyses.

**Results:**

Calpeptin reduced EV secretion and arachidonic acid proportions in adipocytes, while also perturbing key metabolic pathways, including those of the dietarily essential polyunsaturated FAs (PUFAs). Potential biomarker candidates associated with calpeptin included C20–22 PUFAs (adipocytes) and 23:0 (EVs). Both PA and the secretome from calpeptin-treated adipocytes induced pro-inflammatory responses in IHHs.

**Conclusion:**

The findings suggest that calpeptin may modulate adipocyte lipid metabolism and EV secretion, with associated inflammatory responses in hepatocytes likely mediated by adipocyte‑derived secreted factors. These observations warrant further investigation into the potential adverse effects of calpeptin in the context of metabolic diseases.

**Supplementary Information:**

The online version contains supplementary material available at 10.1007/s00011-026-02280-z.

## Introduction

Calpeptin is a calpain inhibitor with therapeutic potential for various inflammatory and fibrotic diseases. Calpain inhibitors are compounds that block the activity of calpains—calcium-dependent cysteine proteases involved in a wide range of cellular processes, including cytoskeletal remodeling, signal transduction, and apoptosis [1, 2]. Calpeptin is currently investigated for its therapeutic effects in several conditions, such as acute kidney injury [3], pancreatic cancer [4], pulmonary fibrosis [5], and neurodegenerative diseases [6] that involve calpain-mediated pathways.

Calpeptin has induced anti-inflammatory effects in various experimental models by reducing the levels of pro-inflammatory cytokines and suppressing inflammasome activation [3, 7]. In murine adipose tissue (AT), calpain inhibition by calpastatin overexpression has decreased adipocyte apoptosis, macrophage accumulation, and fibrosis, suggesting a role in the modulation of obesity-induced AT inflammation [8]. Calpains regulate key signaling pathways, such as responses to insulin and glucose uptake [9, 10]. For example, they influence actin reorganization, essential for the insulin-stimulated glucose transporter type 4 translocation to the plasma membrane, in murine 3T3-L1 adipocytes [9]. Recently, calpeptin was shown to reduce the expression and secretion of key inflammatory cytokines and chemokines in human adipocytes [11].

Calpeptin was previously found to decrease the secretion of extracellular vesicles (EVs) from various cell types [12], including the release of phosphatidylserine-exposing EVs from human platelets [13]. Calpeptin also previously reduced EV secretion from human adipocytes and altered the expression of proteins involved in EV secretory pathways [11]. While it exerted anti-inflammatory effects on adipocytes, there were also potentially adverse effects on insulin signaling, adiponectin expression, and markers of oxidative stress. The ability of calpeptin to modulate EV release may have significant therapeutic implications, making it a potential tool for both mechanistic studies and therapeutic intervention.

As calpeptin is explored as a treatment for obesity-related disorders, it is crucial to examine its effects on human AT, a recognized target of its action, with particular emphasis on lipid metabolism. This is especially relevant due to the potential health benefits and risks related to the effects of calpeptin on the balance between inflammatory and anti-inflammatory fatty acids (FAs). Moreover, since AT-derived EVs and their cargo contribute to AT–liver crosstalk [14], changes in adipocyte FA composition and secretory profiles may have downstream effects on hepatic inflammatory status. We hypothesized that (*i*) calpeptin would decrease the proportions of pro-inflammatory FAs in adipocytes and their EVs, (*ii*) calpeptin would increase the percentages of anti-inflammatory FAs that serve as precursors for pro-resolving lipid mediators (LMs), and (*iii*) calpeptin-induced changes in the adipocyte secretome would suppress inflammatory responses in hepatocytes, revealing a potential mechanism of AT–liver communication in metabolic diseases.

## Materials and methods

### Adipocyte culture and calpeptin treatment

**Fig. 1:**
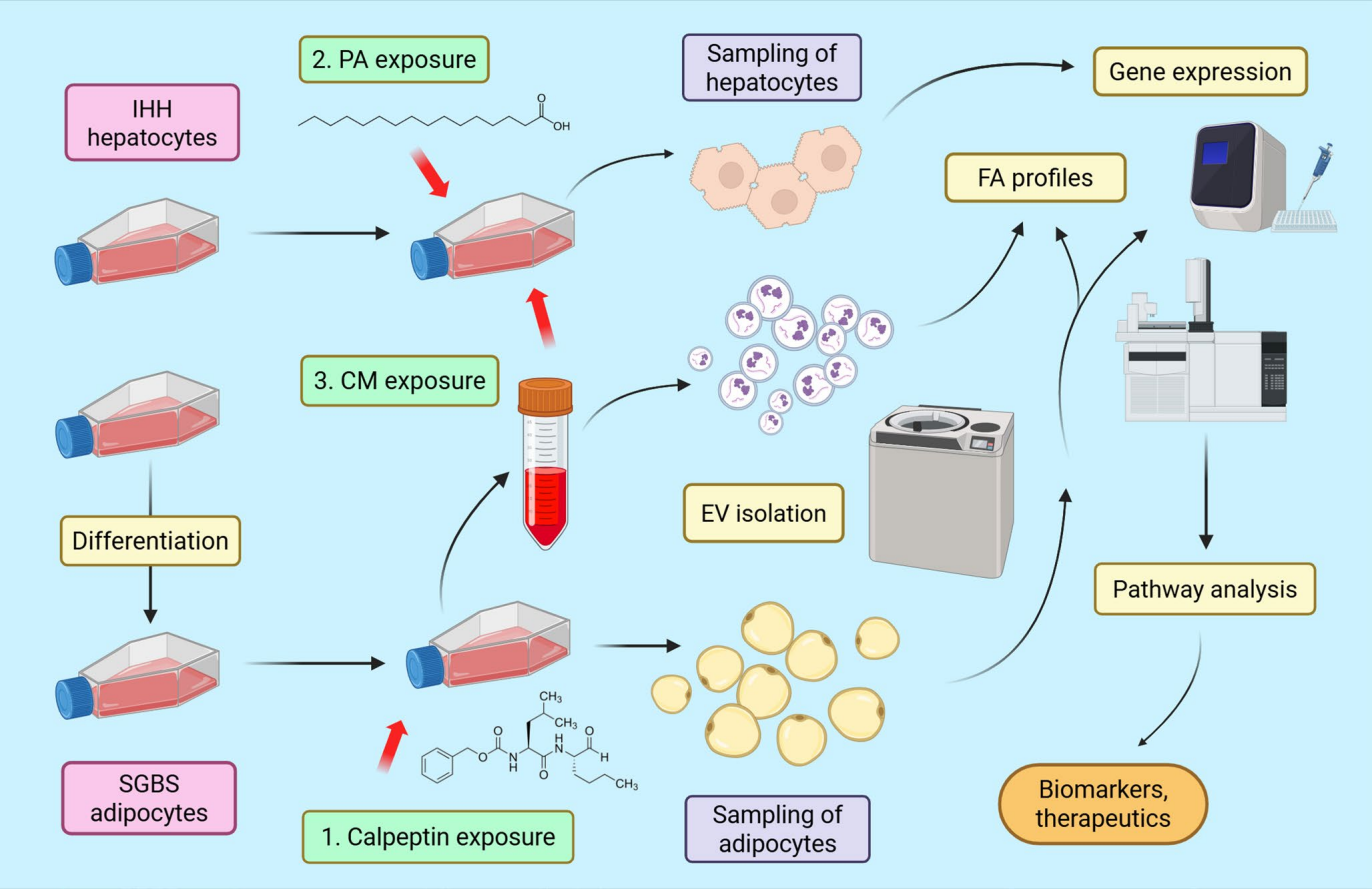
Illustration summarizing the study protocol. Created in BioRender.com, Mustonen, A.-M. (2026) https://BioRender.com/grx2x7y, CM = conditioned medium, EV = extracellular vesicle, FA = fatty acid, IHH = immortalized human hepatocyte, PA = palmitic acid (16:0), SGBS = Simpson-Golabi-Behmel Syndrome

Human Simpson-Golabi-Behmel Syndrome (SGBS) preadipocytes were kindly donated by Maija Vaittinen, Jussi Pihlajamäki (University of Eastern Finland, Kuopio, Finland), and Martin Wabitsch (Ulm University Medical Center, Ulm, Germany). They represent the most utilized cell model for human adipogenesis and adipocyte biology, including investigations on insulin sensitivity, inflammatory responses, and adipokine secretion [15]. SGBS preadipocytes were cultured, differentiated, and exposed to calpeptin as outlined previously [11] (Fig. 1). In brief, SGBS adipocytes were treated for 24 h with EV-depleted 3% 3FC medium containing 25 µM or 50 µM calpeptin (C8999, Sigma-Aldrich, St. Louis, MO, USA). Calpeptin concentrations of similar magnitude were also used in previous experiments to inhibit EV release [12], supporting their suitability for mechanistic in vitro studies. Before culture, EVs were depleted from fetal bovine serum by 110,000 × *g* ultracentrifugation for 16 h at +4 °C [16], followed by sterile filtration through 0.22 μm syringe filters. Cell viability, assessed by confocal microscopy and propidium iodide staining, was not affected by calpeptin exposure [11].

To verify that calpeptin treatment induced calpain inhibition in SGBS adipocytes, the protein expressions of calpain-1 and calpain-2 were determined by Western blotting, as outlined previously [11]. Calpeptin treatment led to expected reductions in calpain protein expression: at 25 µM, calpeptin reduced calpain-1 (80 kDa) by 50%, calpain-2 (80 kDa) by 85%, and calpain-2 (28 kDa) by 40%, and at 50 µM calpeptin, the reductions were 77, 56, and 88%, respectively [11]. The observed decreases in calpain-1 and calpain-2 protein expression indicate that the selected calpeptin concentrations were functionally appropriate to be used in our SGBS adipocyte culture.

### EV isolation and quality control

Conditioned medium (CM) from SGBS adipocytes was filtered through 5 μm syringe filters to remove cell debris, and EVs were isolated by standard ultracentrifugation (Beckman Optima L-90K, Beckman Coulter Inc., Brea, CA, USA). CM was first centrifuged at 10,000 × *g* for 90 min at +4 °C. The resulting supernatant was then subjected to ultracentrifugation at 110,000 × *g* for 90 min at +4 °C. The pellets obtained from both the 10,000 × *g* and 110,000 × *g* centrifugation steps were combined and resuspended in sterile-filtered phosphate-buffered saline (PBS) and stored at −80 °C.

The purity and quality of the EV isolates for FA analysis were thoroughly verified using transmission electron microscopy (JEOL JEM 2100F, Jeol Ltd., Tokyo, Japan), confocal microscopy (Zeiss Axio Observer inverted microscope with a Zeiss LSM 800 confocal module, Carl Zeiss Microimaging GmbH, Jena, Germany), and Western blotting, as described previously [11]. Careful assessment confirmed that the EVs exhibited characteristic cup-shaped morphology and expressed common EV markers, including tetraspanins CD9 and CD63, tumor susceptibility 101, β-actin, programmed cell death 6-interacting protein, and adiponectin. The comprehensive evaluation also excluded cross-contamination with cellular organelles, as evidenced by the absence of calnexin. These EV data have been reported in our previous paper [11].

Particle counts and size distribution were determined by nanoparticle tracking analysis with the Nanoparticle Tracking Analyzer (Malvern Instruments Ltd., Malvern, UK). Based on these previously published data [11], EV secretion significantly decreased at 25 and 50 µM calpeptin (–30% and −26%, respectively). The size distribution of secreted EVs was not affected by calpeptin treatment.

### Hepatocyte culture and CM treatment

To investigate how the SGBS adipocyte secretome influences liver cells, immortalized human hepatocytes (IHHs) were treated with CM from calpeptin-treated SGBS adipocytes, as previously described [17]. This cell line produces key inflammatory cytokines in response to lipotoxic stress, making it a relevant in vitro model for studying inflammatory pathways in the context of liver steatosis [18]. IHHs, kindly donated by Vesa Olkkonen (Minerva Foundation, Helsinki, Finland), were cultured in 10% William’s E growth medium supplemented with 1% penicillin/streptomycin and 1% L-glutamine. To induce a pre-existing inflammatory state [19], cells were exposed to 400 µM palmitic acid (PA, 16:0) in EV-depleted IHH growth medium for 24 h. PA was complexed with 10% bovine serum albumin (BSA) in EV-depleted PBS at a PA:BSA molar ratio of 4:1. CM from SGBS adipocytes treated with calpeptin (0, 25, or 50 µM for 24 h) were diluted 1:1 with EV-depleted IHH growth medium and applied to IHHs, either with or without a prior PA treatment. The cells were incubated with the diluted CM for 6 h, after which samples were collected and stored at −80 °C.

### FA analysis

The FA profiles of SGBS adipocytes (control: *n* = 4, calpeptin 25 µM: *n* = 3, 50 µM: *n* = 3) and EV fractions isolated from CM after 24-h culture (control: *n* = 4, calpeptin 25 µM: *n* = 3, 50 µM: *n* = 3) were analyzed by gas chromatography–mass spectrometry [20]. Excess water was removed from the samples by a stream of N_2_, followed by transmethylation in 1% v/v H_2_SO_4_ in MeOH under a N_2_ atmosphere. This step converted both free and lipid-bound FAs into FA methyl esters, and the alkenyl chains of plasmalogen phospholipids (PLs) into dimethyl acetals (DMAs), which were extracted with hexane and subsequently dried using anhydrous Na_2_SO_4_. The structures were identified using electron impact mass spectra recorded by the Shimadzu GCMS-QP2010 Ultra with a mass-selective detector (Shimadzu, Kyoto, Japan). The quantitative composition was determined by the Shimadzu GC-2010 Plus gas chromatograph equipped with a flame ionization detector, offering a wide linear range of detector response. Chromatographic peaks were manually integrated, and the results expressed as mol-% of total lipid side chains in SGBS adipocytes or CM-derived EVs.

### RNA-sequencing and real-time quantitative PCR (RT-qPCR)

Gene expression analyses of adipocytes and hepatocytes have been described in detail in our previous publication and thesis where part of the data has been reported [11, 17]. In brief, RNA-sequencing of SGBS adipocytes (*n* = 3 for both control and 50 µM calpeptin) was performed by CeGaT GmbH (Tübingen, Germany), using the SMART-Seq Stranded Kit (Takara Bio, Kusatsu, Shiga, Japan) for RNA library preparation. For RT-qPCR, total RNA was extracted (*n* = 3–4 per treatment) using standard procedures, followed by cDNA synthesis with the Verso cDNA Synthesis Kit (Thermo Fisher Scientific, Vilnius, Lithuania). RT-qPCR was carried out using the LightCycler 480 SYBR Green I Master (Roche Diagnostics, Espoo, Finland) with hypoxanthine phosphoribosyltransferase 1 (*HPRT1*) as the reference gene for the adipocytes and glyceraldehyde-3-phosphate dehydrogenase (*GAPDH*) for the hepatocytes. Data are expressed as fold changes relative to the control group.

### Statistical and pathway analyses

This article presents novel FA profiling data from adipocytes and their EVs, whereas data on adipocyte viability, EV secretion, EV characterization, calpain protein expression, and adipocyte and IHH gene expression were largely derived from previously reported datasets [11, 17] and are incorporated only as background and supporting evidence. Accordingly, the statistical analyses described below place emphasis on the newly generated FA profiling data.

Conventional statistical analyses were performed with the IBM SPSS *v*27 software (IBM, Armonk, NY, USA). The Kruskal–Wallis test was utilized to compare three study groups (control, 25 µM, and 50 µM calpeptin), while the Mann–Whitney U test was applied for comparisons between two groups. A *p*-value < 0.05 was considered statistically significant, and the results were presented as the mean ± standard error (SE). For the RNA-sequencing data, differential expression analysis was performed in R (*v*4.3.2) using the limma (*v*3.58.1) and edgeR (*v*4.0.15) packages. Transcript abundance estimates were generated, and the abundance files were processed using the tximport package in R to obtain gene-level counts. Genes with a false discovery rate < 0.05 were considered differentially expressed (DEGs). To limit the number of false-positive findings, all DEGs were examined manually based on the signal tracks using the Integrative Genomics Viewer (https://igv.org/*).*

The MetaboAnalyst software *v*6.0 (https://www.metaboanalyst.ca*)* was utilized to perform data normalization, processing, and pathway analysis for the FA data [21]. The values were normalized by the median, followed by log transformation. FAs that could have potential as biomarkers of calpeptin effects were selected based on variable importance in projection (VIP) values and loadings plots from the principal component analysis (PCA). The performance of each biomarker candidate was evaluated by calculating the area under the receiver operating characteristic (ROC) curve (AUC), along with sensitivity and specificity.

The obtained DEGs were imported into the Search Tool for the Retrieval of Interacting Genes/Proteins (STRING) *v*12.0 [22], a comprehensive online resource (https://string-db.org/) designed to evaluate known and predicted protein–protein interactions (PPIs). The research species was set to *Homo sapiens*, the minimum required interaction score of 0.400 (medium confidence) was selected, and PPI network diagrams were constructed for control and 50 µM calpeptin-treated SGBS adipocytes. Enrichment analyses included Gene Ontology (GO) classifications for biological processes and molecular functions, as well as pathway enrichment based on the Kyoto Encyclopedia of Genes and Genomes (KEGG) database.

## Results

### Selective incorporation of FAs into EVs

**Fig. 2:**
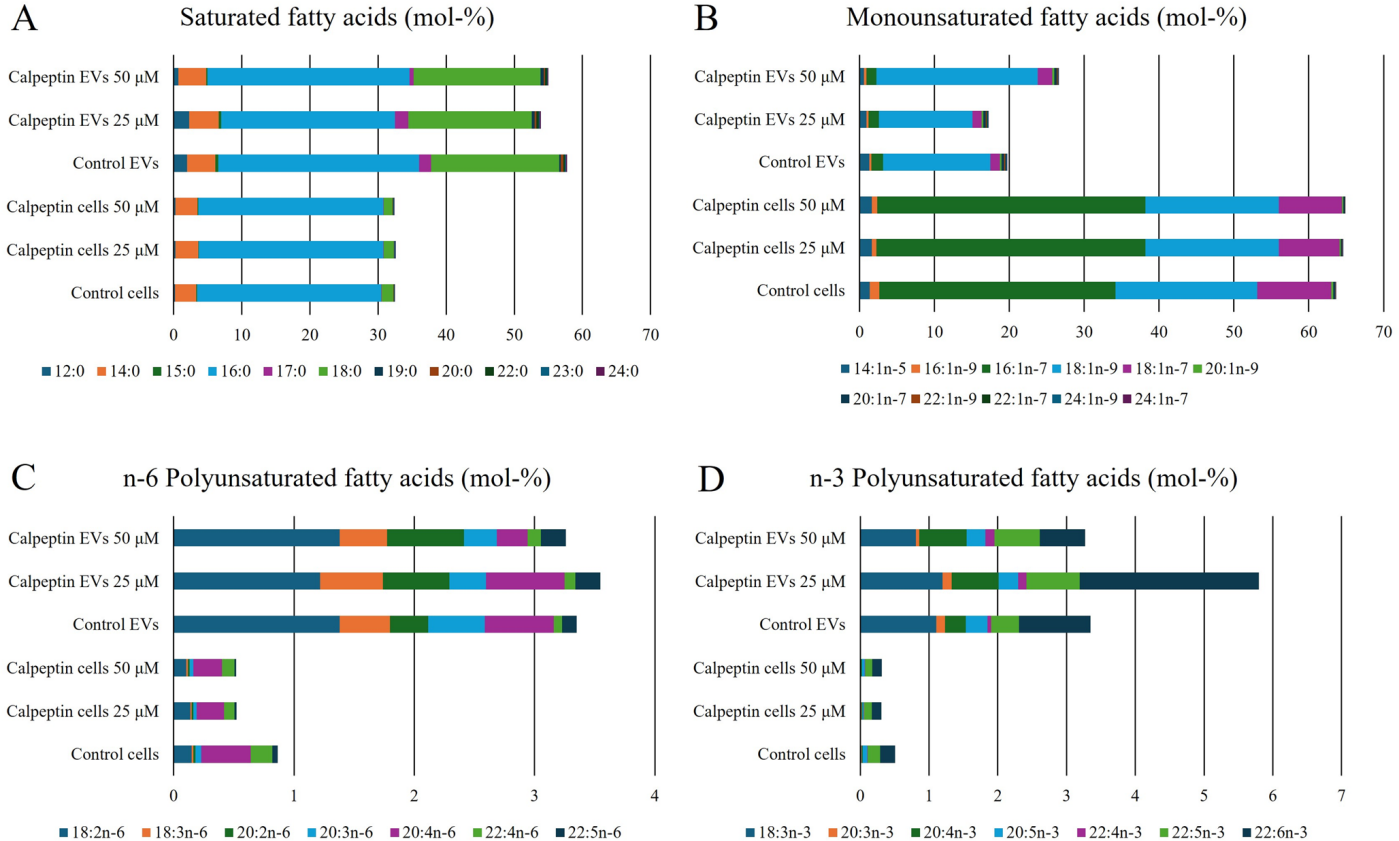
Average proportions (mol-%) of selected individual fatty acids (FAs) in control and calpeptin-treated (25 or 50 µM) Simpson-Golabi-Behmel Syndrome adipocytes (cells) and their extracellular vesicles (EVs). Panel (**A**) represents saturated FAs, panel (**B**) monounsaturated FAs, panel (**C**) n-6 polyunsaturated FAs, and panel (**D**) n-3 polyunsaturated FAs

Compared to the donor SGBS adipocytes, the FA profiles of EVs were characterized by higher percentages of total saturated FAs (SFAs) as well as n-6 and n-3 polyunsaturated FAs (PUFAs), while the proportions of total monounsaturated FAs (MUFAs) were lower in EVs (Fig. 2, Supplementary Table S1). Several individual SFAs, encompassing both straight-chain as well as *iso* and *anteiso* branched-chain FAs across chain lengths C12–24, were higher in EVs (Mann–Whitney U test, *p* < 0.001–0.006). In addition, the proportions of DMA 16:0 and DMA 18:0 were higher, and due to the low MUFA level, the overall unsaturated FA (UFA)/SFA ratio was lower in EVs (Mann–Whitney U test, *p* < 0.001–0.010).

Among individual MUFAs, EVs exhibited lower proportions of selected C14–18 FAs compared to cells, whereas several other MUFAs, also including longer-chain C22–24 forms, were present at higher proportions (Mann–Whitney U test, *p* < 0.001–0.003). The percentages of DMA 18:1n-9 and DMA 18:1n-7 were also higher in EVs (Mann–Whitney U test, *p* < 0.001). Several n-3 PUFAs, ranging from 18:3n-3 to 22:5n-3 and 22:6n-3, along with the n-3/n-6 PUFA ratio, were higher in EVs compared to cells (Mann–Whitney U test, *p* < 0.001–0.019). In contrast, the ∆5-desaturation index and product/precursor ratios of n-3 PUFAs were lower in EVs (Mann–Whitney U test, *p* < 0.001–0.001).

Several n-6 PUFAs, from 18:2n-6 to 20:3n-6 and 22:5n-6, were elevated in EVs relative to SGBS adipocytes (Mann–Whitney U test, *p* < 0.001–0.002). The ∆6-desaturation index of n-6 PUFAs was also higher in EVs, whereas the ∆5-desaturation index and the product/precursor ratios of n-6 PUFAs were lower compared to cells (Mann–Whitney U test, *p* < 0.001–0.034). The proportions of total DMAs, 20:2n-9, 22:2n-9, and 22:3n-9 were higher, and those of 20:3n-9 lower in EVs (Mann–Whitney U test, *p* < 0.001–0.016).

**Fig. 3:**
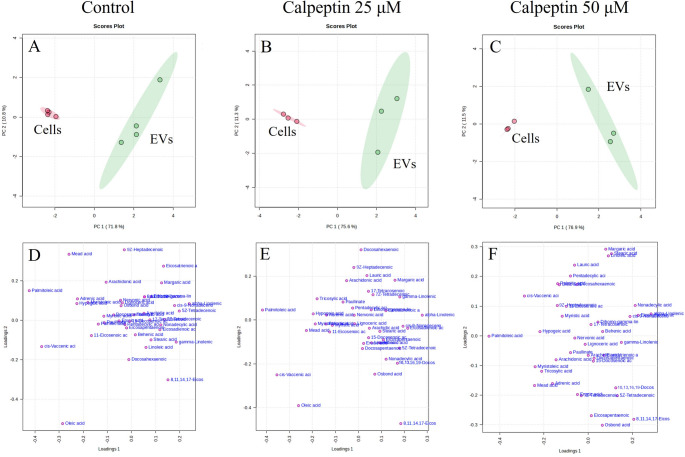
Principal component analysis (PCA) of Simpson-Golabi-Behmel Syndrome adipocytes (Cells) and their extracellular vesicles (EVs) by MetaboAnalyst based on the fatty acid (FA) profiles. Panels (**A**–**C**) show PCA scores plots and panels (**D**–**F**) display loadings plots of individual FAs marked by trivial names listed in Supplementary Table S2, PC = principal component, red = cells, green = EVs

**Fig. 4:**
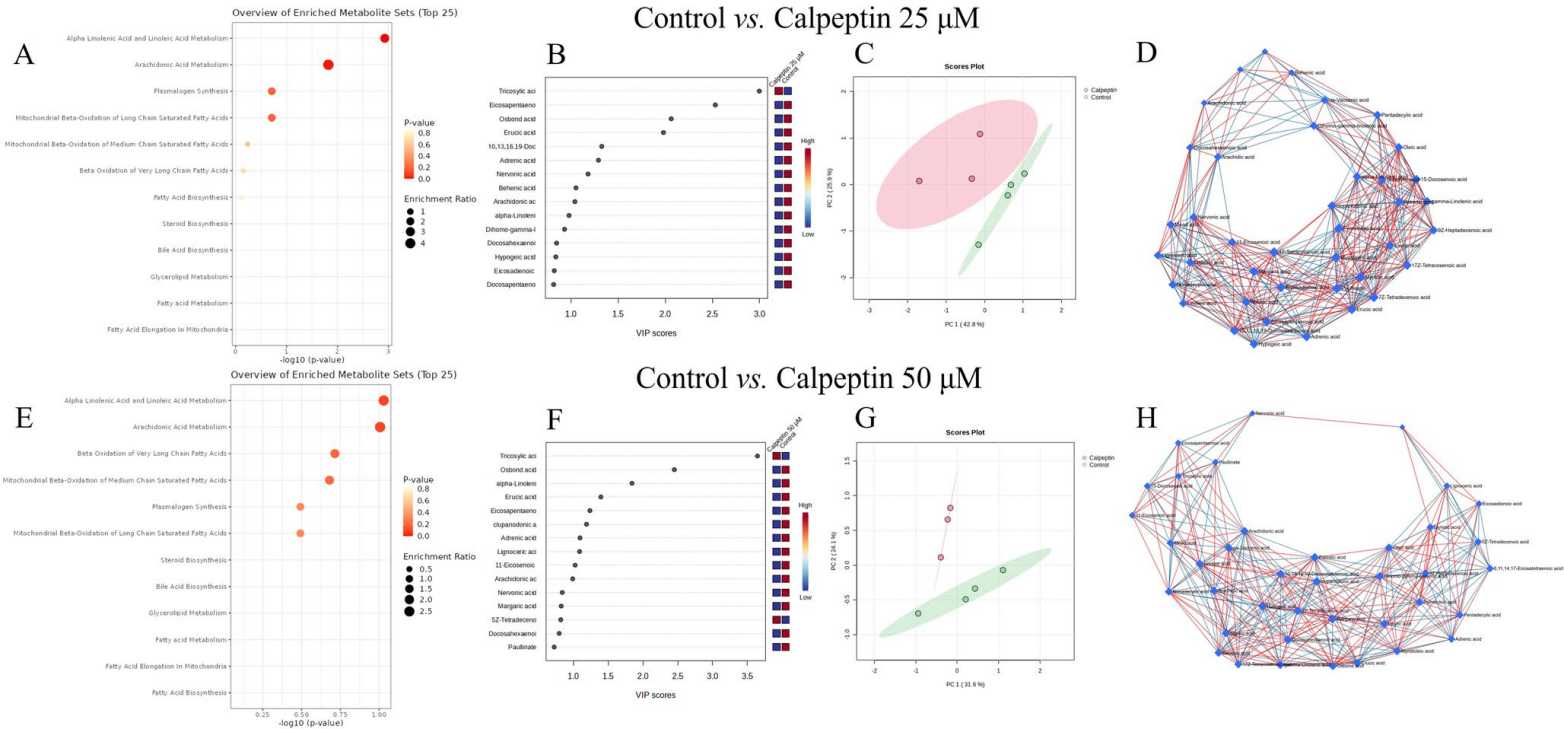
Enriched metabolite sets (panels** A**,** E**), variable importance in projection (VIP) scores (**B**,** F**), principal component analysis scores plots (**C**, **G**), and correlations of metabolic pathways of fatty acids (FAs) (**D**,** H**) in Simpson-Golabi-Behmel Syndrome adipocytes treated with calpeptin, analyzed using MetaboAnalyst. Panels (**A**–**D**) represent 25 µM calpeptin and control and panels (**E**–**H**) 50 µM calpeptin and control. FAs are marked by trivial names listed in Supplementary Table S2. In panels D and H, blue connection lines represent negative correlations, whereas red connection lines represent positive correlations. Blue diamonds represent metabolites linked to pathways related to the input metabolites, forming clusters. PC = principal component, green = control, red = calpeptin-treated

**Fig. 5:**
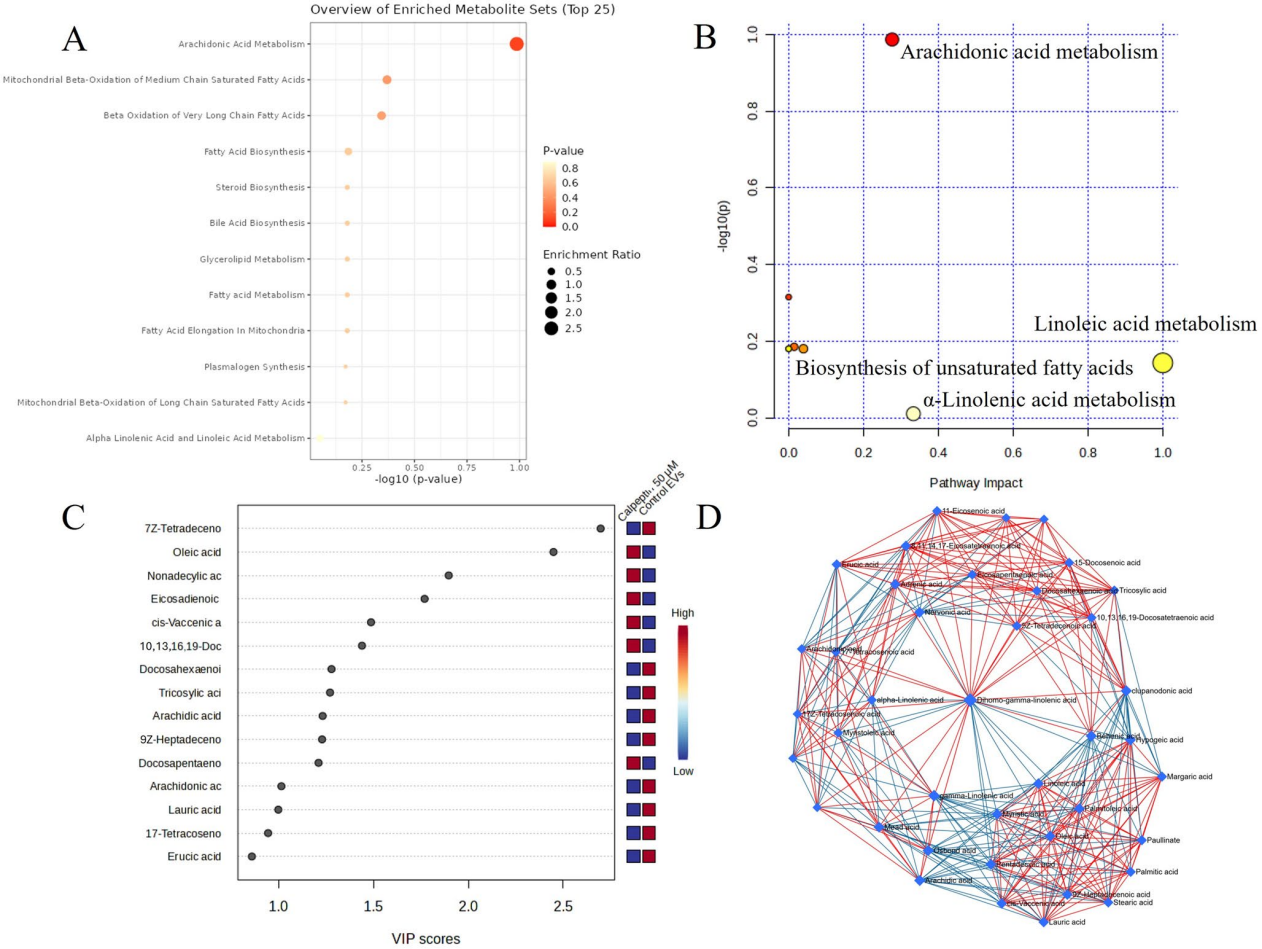
Enriched metabolite sets (panel** A**), summary of metabolic pathway analysis (**B**), variable importance in projection (VIP) scores (**C**), and correlations of metabolic pathways of fatty acids (FAs) (**D**) in extracellular vesicles (EVs) derived from Simpson-Golabi-Behmel Syndrome adipocytes treated with 50 µM calpeptin and controls, analyzed using MetaboAnalyst. FAs are marked by trivial names listed in Supplementary Table S2. In panel D, blue connection lines represent negative correlations, whereas red connection lines represent positive correlations. Blue diamonds represent metabolites linked to pathways related to the input metabolites, forming clusters

In the PCA, principal components (PCs) 1 and 2 clearly separated the cell groups from the EV groups across calpeptin concentrations 0, 25, and 50 µM (Fig. 3A–C). The individual FAs responsible for the separation included, e.g., 16:1n-7, 18:1n-7, and 18:1n-9 MUFAs, and are represented in Fig. 3D–F. In Figs. 3, 4 and 5, FAs are labeled with their trivial names, as required by the MetaboAnalyst platform; these are listed in Supplementary Table S2. Based on the pathway analysis by MetaboAnalyst, the most enriched metabolite sets were 18:3n-3 and 18:2n-6 metabolism, 20:4n-6 metabolism, steroid biosynthesis, bile acid biosynthesis, glycerolipid metabolism, FA metabolism, FA elongation in mitochondria, plasmalogen synthesis, mitochondrial β-oxidation of long-chain SFAs, and FA biosynthesis (data not shown). In addition, 18:3n-3 metabolism, 20:4n-6 metabolism, 18:2n-6 metabolism, and the biosynthesis of UFAs showed the highest statistical significance and/or pathway impact between the cells and EVs in the metabolic pathway analysis.

### Effects of calpeptin on FAs in SGBS adipocytes

The percentages of 20:4n-6 and DMA 16:0 were reduced in SGBS adipocytes following treatment with both 25 µM and 50 µM calpeptin (Kruskal–Wallis test, *p* = 0.038; Supplementary Table S1). The 50 µM treatment also decreased the proportions of 18:1n-5, 22:3n-9, 22:5n-6, total n-6 PUFAs, and total PUFAs compared to controls (Kruskal–Wallis test, *p* = 0.035–0.046). Additionally, there was a trend toward lower proportions of 20:5n-3 and 22:4n-6 at 25 µM calpeptin, 18:3n-3 and 22:5n-3 at 50 µM calpeptin, and n-3 PUFA sum at both concentrations (Kruskal–Wallis test, *p* = 0.062–0.082).

According to the metabolite set enrichment analysis of SGBS adipocytes, 18:3n-3 and 18:2n-6 metabolism, 20:4n-6 metabolism, plasmalogen synthesis, mitochondrial β-oxidation of long-chain SFAs and, to a lesser extent, mitochondrial β-oxidation of medium-chain SFAs and β-oxidation of very-long-chain FAs were disturbed by 25 µM calpeptin (Fig. 4A). These same pathways were also affected at 50 µM calpeptin, albeit in a slightly different order of importance and with lower statistical significance (Fig. 4E). In the metabolic pathway analysis, the biosynthesis of UFAs, 20:4n-6 metabolism, 18:2n-6 metabolism, and 18:3n-3 metabolism showed the highest significance and/or pathway impact at 25 µM calpeptin. At 50 µM, 18:3n-3 metabolism, 18:2n-6 metabolism, and 20:4n-6 metabolism exhibited the highest statistical significance and/or pathway impact.

FAs 23:0, 20:5n-3, 22:5n-6, 22:1n-9, 22:4n-3, 22:4n-6, 24:1n-9, 22:0, 20:4n-6, 18:3n-3, 20:3n-6, and 22:6n-3 showed the highest VIP scores for 25 µM calpeptin (Fig. 4B), whereas mostly the same FAs, i.e., 23:0, 22:5n-6, 18:3n-3, 22:1n-9, 20:5n-3, 22:5n-3, 22:4n-6, 24:0, 20:1n-9, 20:4n-6, and 24:1n-9, had the highest VIP scores for 50 µM calpeptin (Fig. 4F). In the PCA, PCs 1 and 2 clearly separated the control samples from both the 25 µM and 50 µM samples, indicating perturbations in cellular FA metabolism by calpeptin exposure (Fig. 4C, G). The separating variables were largely consistent with those showing the highest VIP scores, with additional contributors including 19:0, 20:2n-6, 16:1n-9, and 18:3n-6, among others.

The ROC analysis for 25 µM calpeptin showed that 18:3n-3, 20:1n-9, 20:4n-6, 20:5n-3, 22:1n-9, 22:4n-6, 22:5n-6, and 22:5n-3 had the greatest biomarker potential for calpeptin effects, with an AUC of 1.000 and both sensitivity and specificity of 1.000. Similarly, 18:3n-3, 20:1n-9, 20:4n-6, 22:4n-6, 22:5n-6, and 22:5n-3 demonstrated the highest biomarker potential for the 50 µM calpeptin group, also with an AUC of 1.000, sensitivity of 1.000, and specificity of 1.000; however, given the small sample size, these findings should be considered preliminary and will require validation in larger studies. Correlations of metabolic pathways between individual FAs in response to 25 and 50 µM calpeptin are presented as interactive networks in Fig. 4D, H. Red edges between FAs indicate positive correlations, whereas blue edges represent antagonistic relationships, potentially reflecting metabolic competition or pathway divergence. Network analysis revealed structural differences between adipocyte groups, as with calpeptin exposure the networks became progressively denser, displaying a greater number of edges connecting FAs.

### Effects of calpeptin on FAs in SGBS adipocyte-derived EVs

In the EVs from SGBS adipocytes, the proportion of 20:0 decreased following exposure to 50 µM calpeptin (Kruskal–Wallis test, *p* = 0.030; Supplementary Table S1). According to the metabolite set enrichment analysis of control and 25 µM calpeptin EV samples, the top enriched metabolite sets included steroid biosynthesis, bile acid biosynthesis, glycerolipid metabolism, FA metabolism, FA elongation in mitochondria, plasmalogen synthesis, and mitochondrial β-oxidation of long-chain SFAs (Supplementary Fig. S1). When control EVs were compared to EVs from 50 µM calpeptin-treated samples, the top pathways were 20:4n-6 metabolism, mitochondrial β-oxidation of medium-chain SFAs, β-oxidation of very-long-chain FAs, FA biosynthesis, steroid biosynthesis, bile acid biosynthesis, glycerolipid metabolism, FA metabolism, FA elongation in mitochondria, plasmalogen synthesis, and mitochondrial β-oxidation of long-chain SFAs (Fig. 5A). However, the individual pathways in the metabolite set enrichment analyses did not reach statistical significance and, at this stage, should be interpreted as exploratory.

18:2n-6 metabolism, glycosylphosphatidylinositol-anchor biosynthesis, 18:3n-3 metabolism, and 20:4n-6 metabolism ranked highest in the pathway enrichment/pathway topology analysis of EVs from 25 µM calpeptin-treated adipocytes compared to control EVs (Supplementary Fig. S1). For 50 µM calpeptin EVs, 20:4n-6 metabolism, 18:2n-6 metabolism, 18:3n-3 metabolism, and the biosynthesis of UFAs had the highest enrichment and/or impact scores (Fig. 5B) but, again, these pathways did not show statistically significant *p*-values and, for this reason, these results should be considered as hypothesis‑generating. In the PCA, PCs 1 and 2 did not distinguish the 25 µM or 50 µM calpeptin-treated EV groups from the controls, while the partial least squares discriminant analysis clearly separated the 50 µM calpeptin group and the control EVs.

The FAs with the highest VIP scores showed partial overlap between the two dose groups when compared with control EVs, although their order of importance differed. For 25 µM calpeptin, the FAs with the highest VIP scores included 20:2n-6, 22:4n-3, 19:0, 22:5n-3, 20:3n-3, 20:3n-6, 22:1n-7, 23:0, 24:1n-9, 20:0, and 22:6n-3 (Supplementary Fig. S1). For 50 µM calpeptin, the top contributors were 14:1n-7, 18:1n-9, 19:0, 20:2n-6, 18:1n-7, 22:4n-3, 22:6n-3, 23:0, 20:0, 17:1n-8, 22:5n-3, and 20:4n-6 (Fig. 5C). Network analysis of FA relationships showed an increase in network density with calpeptin exposure, characterized by more numerous associations and the emergence of distinct PUFA hubs, such as 20:3n-6 in the 50 µM calpeptin dataset (Fig. 5D).

The ROC analysis for EVs from 25 µM calpeptin-treated adipocytes showed that 23:0 had the greatest biomarker potential, with an AUC, specificity, and sensitivity of 1.000. It was followed by 20:2n-6 and 22:4n-3, both with an AUC value of 0.833, specificity of 1.000, and sensitivity of 0.750. Additional potential biomarker candidates included 22:5n-3, 14:1n-9, and 16:1n-9 with AUCs of 0.750–0.833, specificities of 0.750–1.000, and sensitivities of 0.670–0.750. For EVs from 50 µM calpeptin-treated adipocytes, 19:0 and 23:0 had the highest AUC, specificity, and sensitivity (all 1.000), followed by 14:1n-7, 20:4n-6, and 20:0 (AUC: 0.833–0.917, specificity: 0.670–1.000, sensitivity: 0.750–1.000). However, the limitations related to the small sample size must, again, be taken into account, and these findings will require validation in larger studies.

### Effects of calpeptin on gene expression in adipocytes and hepatocytes

**Fig. 6:**
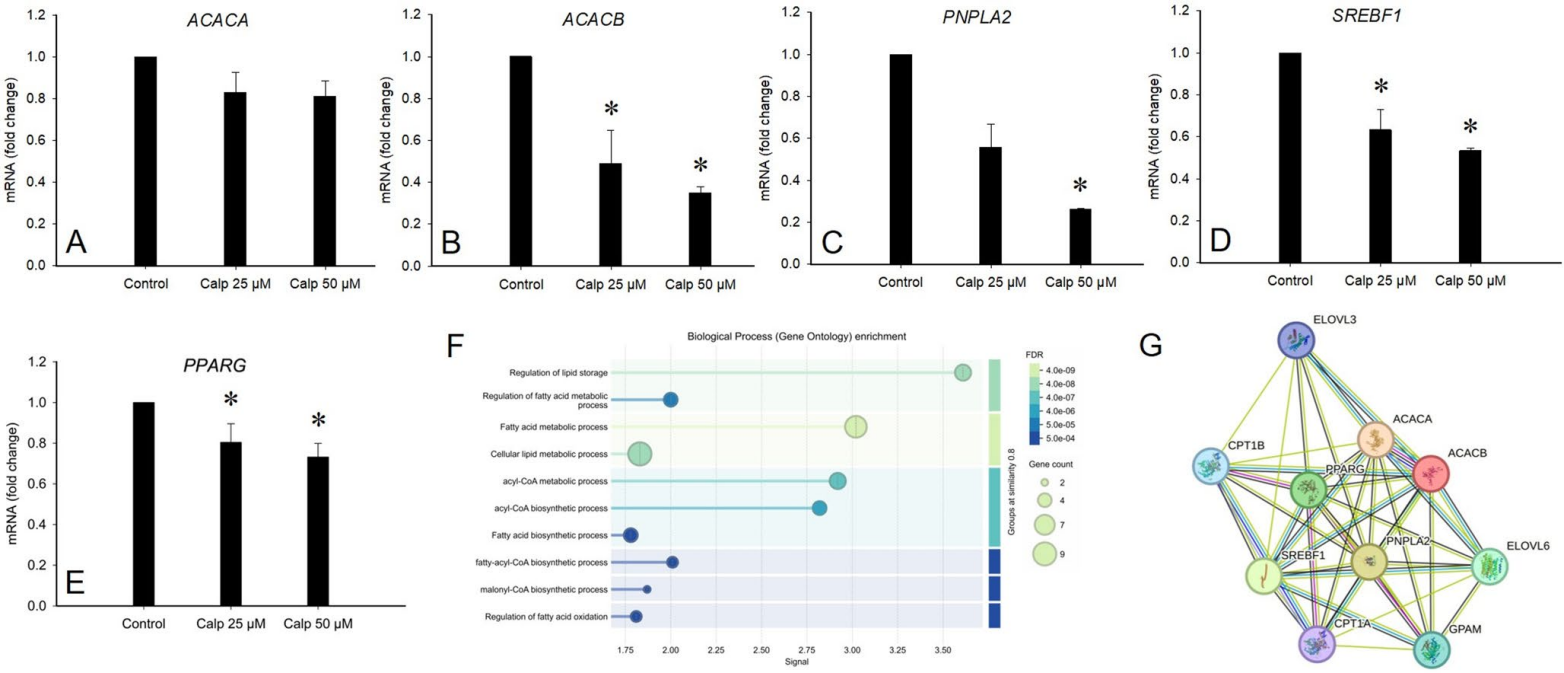
The mRNA expression of lipid metabolism-related genes *ACACA*, *ACACB*, *PNPLA2*, *SREBF1*, and *PPARG* in Simpson-Golabi-Behmel Syndrome (SGBS) adipocytes treated with 0, 25, or 50 µM calpeptin (Calp) (panels **A–E**, mean + SE), *n* = 3–4 per treatment, * *p* = 0.018 for *ACACB*, *p* = 0.024 for *PNPLA2*, *p* = 0.021 for *SREBF1*, and *p* = 0.016 for *PPARG* compared to control (Kruskal–Wallis test). Gene Ontology biological processes, scaled according to false discovery rate (FDR) cutoff values to indicate the significance after correcting for multiple testing to avoid false positives (panel** F**), and protein–protein interaction (PPI) network (panel** G**), based on the Search Tool for the Retrieval of Interacting Genes/Proteins analysis of differentially expressed genes between control and 50 µM calpeptin-treated SGBS adipocytes. Network (panel** G**) nodes represent proteins, and edges represent PPIs. Colored nodes indicate query proteins and first shell of interactors. The colored edges connecting proteins indicate known interactions in turquoise (from curated databases) and purple (experimentally determined). Predicted interactions are shown in green (gene neighborhood), red (gene fusions), and dark blue (gene co-occurrence). Yellow color indicates text mining, black co-expression, and light blue protein homology

Based on the DEGs from the RNA-sequencing data, perilipin 1 (*PLIN1*), diacylglycerol O-acyltransferase 2 (*DGAT2*), ELOVL fatty acid elongase 6 (*ELOVL6*), and angiopoietin-like 8 (*ANGPTL8*) were downregulated in SGBS adipocytes exposed to 50 µM calpeptin compared to control cells, while *PLIN4* showed increased expression (adj. *p* = 0.007–0.047; data not shown). The comprehensive list of DEGs identified in the RNA-sequencing analysis is presented in [11]. In RT-qPCR, acetyl-CoA carboxylase beta (*ACACB*), patatin like domain 2, triacylglycerol lipase (*PNPLA2*), sterol regulatory element binding transcription factor 1 (*SREBF1*), and peroxisome proliferator-activated receptor gamma (*PPARG*) were downregulated in SGBS adipocytes at 25 and/or 50 µM calpeptin (Kruskal–Wallis test, *p* = 0.016–0.024), but the expression of *ACACA* did not change significantly due to calpeptin (Fig. 6A–E).

For the DEGs in adipocytes, STRING GO biological processes analysis highlighted regulation of lipid storage, regulation of FA metabolic process, FA metabolic process, cellular lipid metabolic process, acyl-CoA metabolic process, and acyl-CoA biosynthetic process, among other lipid metabolism-related pathways (Fig. 6F). The discovered KEGG pathways included, e.g., FA metabolism, AMPK signaling pathway, glucagon signaling pathway, insulin resistance, and adipocytokine signaling pathway. In the PPI network, there were 10 nodes and 40 edges, the average node degree was 8, the average local clustering coefficient was 0.919, and the PPI enrichment *p*-value was < 1.0 × 10⁻^16^ (Fig. 6G).

**Fig. 7:**
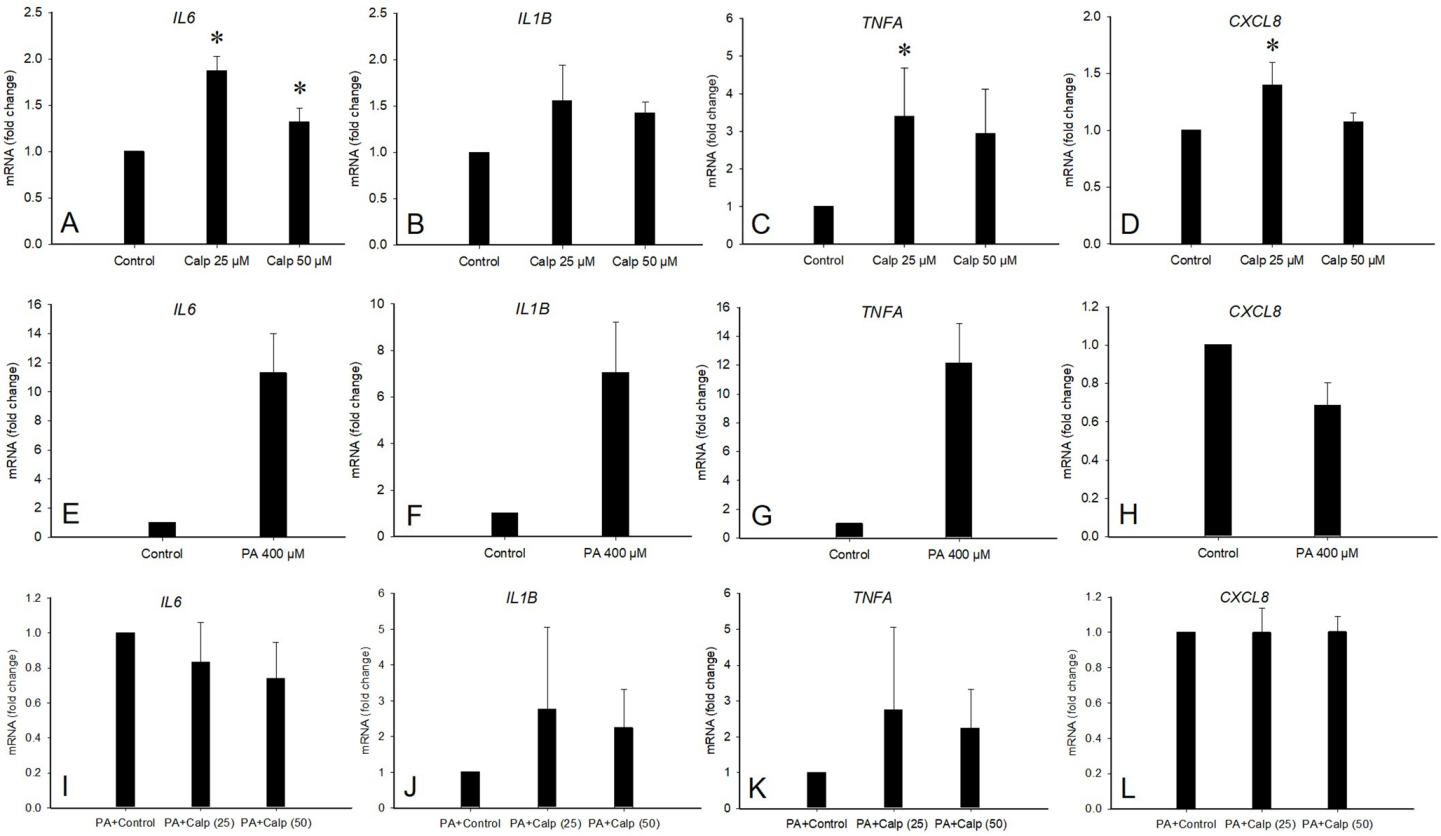
The mRNA expression of inflammatory cytokines *IL6*, *IL1B*, *TNFA*, and *CXCL8* in immortalized human hepatocytes (IHHs) treated with conditioned medium (CM) from calpeptin (Calp) treated Simpson-Golabi-Behmel Syndrome (SGBS) adipocytes (0, 25, or 50 µM) (panels **A–D**), *n* = 4 per treatment, * *p* = 0.029 compared to control (Mann–Whitney U test). The mRNA expression of *IL6*, *IL1B*, *TNFA*, and *CXCL8* in IHHs treated with 0 or 400 µM palmitic acid (PA) (panels **E–H**), *n* = 1 with four replicates, average fold changes were 11.3, 7.1, 12.2, and 0.7, respectively. The mRNA expression of *IL6*, *IL1B*, *TNFA*, and *CXCL8* in IHHs treated with 400 µM PA followed by a treatment with CM from calpeptin-treated SGBS adipocytes (0, 25, or 50 µM) (panels **I–L**), *n* = 3–4 per treatment. Data are presented as mean + SE

Regarding inflammatory crosstalk with hepatocytes, the gene expression of interleukin-6 (*IL6*), tumor necrosis factor alpha (*TNFA*), and C-X-C motif chemokine ligand 8 (*CXCL8*) increased in IHHs exposed to CM derived from SGBS adipocytes treated with 25 and/or 50 µM calpeptin (Mann–Whitney U test, *p* = 0.029), while the expression of *IL1B* or *PPARG* did not change significantly (Fig. 7A–D). The treatment of IHHs with 400 µM PA increased the gene expression of *IL6*, *IL1B*, and *TNFA*, while *CXCL8* was downregulated, but these observations were based on technical replicates only and were not subjected to statistical testing (Fig. 7E–H). The exposure of PA-treated IHHs to CM from calpeptin-treated SGBS adipocytes (0, 25, and 50 µM) did not further amplify inflammatory gene expression (Fig. 7I–L).

## Discussion

Calpains are calcium-dependent cysteine proteases that play crucial roles in regulating a wide range of cellular functions, including insulin signaling and glucose uptake [9, 10]. However, studies investigating calpain function in adipocytes remain limited, and the present experiment addressed this knowledge gap. By exploring the effects of the calpain inhibitor, calpeptin, on adipocyte FA metabolism and downstream hepatocyte inflammation, our findings may contribute to a better understanding of pathways potentially relevant to the pathogenesis and treatment of obesity-related disorders. In short, calpeptin disrupted lipid metabolism in human SGBS adipocytes (*i*) and altered several key metabolic pathways, including the metabolism of the dietarily essential PUFAs (*ii*). Several putative biomarker candidates, such as C20–22 n-6 and n-3 PUFAs (for adipocytes) and 23:0 (for EVs), were identified to be associated with calpeptin treatment (*iii*). Calpeptin induced changes in adipocyte secretory profiles that promoted inflammatory gene expression in hepatocytes (*iv*). The results emphasize the need for comprehensive investigation of calpeptin’s effects on AT and liver metabolism, as well as inter-organ communication and suggest that its therapeutic potential for obesity-related pathologies should be reconsidered.

Based on the current findings, calpeptin could disrupt FA metabolism in human adipocytes, with discernible effects already evident at 25 µM calpeptin. At 50 µM, there were reductions, for instance, in the proportions of 20:4n-6, 22:5n-6, and total n-6 PUFAs. Since n-6 PUFAs are generally considered pro-inflammatory [23], the decreases in their proportions may represent a beneficial effect of calpain inhibition on low-grade inflammation and support our initial hypothesis regarding the anti-inflammatory potential of calpeptin. However, several LMs derived from n-6 PUFAs, such as 1-series prostaglandins from 20:3n-6 and 15-deoxy-∆^12,14^-prostaglandin J_2_ and lipoxin A_4_ from 20:4n-6, possess anti-inflammatory or pro-resolving properties [23], making this issue more complex. We also observed several borderline decreases in the proportions of n-3 PUFAs, precursors for specialized pro-resolving mediators, and stable n-3/n-6 PUFA ratios. In addition, the proportions of individual SFAs—particularly PA, which has been linked to pro-inflammatory effects, e.g., in adipocytes, primary myotubes, and hepatocytes [19, 24, 25]—remained unaffected by calpeptin. These findings highlight that the effects of calpeptin on adipocyte FA composition, especially regarding inflammation, cannot be characterized as purely beneficial or detrimental, supporting our earlier gene and protein expression data from the same cell line [11]. Calpeptin previously disrupted insulin signaling pathways and led to the suppression of adiponectin mRNA and protein expression as well as secretion, supporting the notion of potentially adverse effects on adipocyte metabolism. In addition, our previous results suggested that calpeptin could trigger oxidative stress pathways linked to insulin resistance and inflammation.

FA incorporation from donor adipocytes into EVs was observed to be selective, with accumulation of several SFAs as well as n-3 and n-6 PUFAs. Notably, 18:0 and 18:2n-6 showed increased proportions in EV lipids, whereas the percentages of especially n-7 MUFAs decreased. Among minor FAs, 24:0 and 24:1n-9 were enriched in EVs, presumably as essential components of membrane hexosylceramides and other sphingolipids [26, 27]. Selective sorting of FAs and other lipids into EVs has been previously reported in adipocytes, fibroblast-like synoviocytes, and various other cell lines [20, 28, 29]. The FA composition of EVs can vary significantly depending on the differentiation status of adipocytes, the availability of FAs in the medium, and the anatomical origin of the cultured AT [29, 30]. The exposure of donor cells to PUFAs modifies the EV PL composition [31], potentially affecting the fluidity, curvature, and stability of EVs, their binding to and uptake by recipient cells [28], as well as the transcellular LM synthesis from precursor PUFAs [23, 31].

Enrichment and pathway analyses revealed key metabolic pathways in both SGBS adipocytes and their EVs that may be influenced by calpeptin treatment. These included the metabolism of 20:4n-6, 18:2n-6, and 18:3n-3, as well as plasmalogen synthesis, mitochondrial β-oxidation of long- and medium-chain SFAs, and β-oxidation of very-long-chain FAs. Of individual FAs, 20:4n-6, 22:4n-6, 22:5n-6, 18:3n-3, 22:5n-3, and 20:1n-9 showed the highest discriminative power between control and calpeptin-treated cells, each with an AUC of 1.000. This suggests that their levels were affected by calpain inhibition and that they may serve as useful FA biomarker candidates for calpeptin exposure, warranting further study with a larger sample size. Moreover, the gene expression data from SGBS adipocytes indicate that calpain inhibition affects not only specific FA pathways (β-oxidation, elongation) but also broader aspects of lipid metabolism, including triacylglycerol synthesis, storage, and lipolysis, as further supported by the STRING enrichment analyses. Among the DEGs, *SREBF1* and *PPARG* represent key transcriptional regulators that could mediate calpeptin’s downstream metabolic effects in adipocytes. Although our genetic data yielded partly incongruent results, the net effect of calpeptin exposure could be a reduction in lipid synthesis and adipocyte storage capacity together with dysregulation of normal lipid turnover and droplet stability [32]. In line with this, calpain-mediated proteolytic production of free amino acids in vascular endothelial cells was recently shown to augment obesity-induced hepatic steatosis [33].

Calpains might also influence the composition and asymmetry of EV lipids, as they are known to cleave key lipid-regulatory proteins, including PL flippases [34], enable membrane blebbing [35], and contribute to structural membrane remodeling via annexin cleavage during repair [36]. The resulting annexin^+^ EVs are enriched in phosphatidylserine and/or phosphatidylethanolamine in their outer membrane leaflet. Rearrangement of structural PLs, such as phosphatidylserine, at the plasma membrane can regulate endocytosis and exocytosis [37]. Calpains can also respond to certain PLs, such as phosphatidylinositol 4,5-diphosphate, enhancing their own activation [38]. Based on these findings, it was not surprising that calpain inhibition also had some effects on the EV FA composition and metabolic pathways, and network analysis further supported the hypothesis that calpeptin may influence selective lipid packaging into EVs. While the underlying mechanisms remain to be elucidated in the context of EV lipid regulation, the results suggest an interesting avenue for future research. The observed pathway enrichment trends did not reach statistical significance and should therefore be interpreted as hypothesis‑generating rather than as definitive evidence of pathway activation. Based on the VIP and ROC analyses, the minor SFAs 23:0, 20:0, and 19:0, along with PUFAs 20:2n-6 and 22:4n-3, showed the highest discriminative power between the EVs from control and calpeptin-treated adipocytes, highlighting their potential as biomarker candidates for distinguishing the effects of calpeptin treatment on EV FA profiles. However, these findings should also be interpreted as preliminary and exploratory in nature, pending future validation in larger studies.

To study the crosstalk between adipocytes and hepatocytes, we treated IHHs with the EV-containing secretome from calpeptin-treated SGBS adipocytes and noted a pro-inflammatory response evidenced by increased expression of *IL6*, *TNFA*, and *CXCL8*. Exposure of PA-treated IHHs to adipocyte secretome did not further amplify inflammatory gene expression, which may be partly due to the relatively short duration of CM exposure (6 h vs. 24 h for calpeptin and PA treatments). Our results suggest that calpeptin induces changes in adipocyte secretory profiles that promote inflammation in hepatocytes, possibly via adipokines, cytokines, or EVs harboring lipids, FAs, and LMs. The specific molecular mediators of inflammation remain unidentified and will have to be assessed in future studies; however, our results are consistent with an effect of the adipocyte secretome/CM rather than EVs alone. This finding is relevant, for instance, in metabolic dysfunction-associated steatotic liver disease MASLD (previously known as non-alcoholic fatty liver disease NAFLD) that is driven not only by hepatic factors, but also by AT-derived signaling [39]. Previous studies have shown that hepatic calpain activity is elevated in high-fat diet (HFD)-fed mice, while calpain-1 knockout mice are protected from HFD-induced liver dysfunction via reduced oxidative stress and inflammatory responses [40]. Additionally, calpains may contribute to steatosis by promoting amino acid release from vascular endothelial cells, which fuels *de novo* lipogenesis in hepatocytes [33]. Unlike these previous studies reporting protective, anti-inflammatory effects of calpain inhibition within the liver, our findings demonstrate that modulation of calpain activity in AT may have pro-inflammatory side-effects in the context of metabolic diseases. Although we initially hypothesized that calpeptin would attenuate inflammatory signaling, our findings suggest that modulation of calpain activity in AT may elicit unintended pro-inflammatory responses that could exacerbate metabolic dysfunction. Therefore, rather than supporting a broadly protective role, our data emphasize the need for caution and a comprehensive re-evaluation of calpain inhibition as a potential therapeutic strategy for metabolic diseases.

It is important to acknowledge that the present study has several limitations. The findings should be interpreted with caution due to the small sample size that limits statistical robustness and may have resulted in optimistic estimates of model performance. Accordingly, the reported AUC values should be interpreted as preliminary, pending validation in larger studies. Nevertheless, we employed the SGBS adipocyte cell strain, which is well-characterized and known for its consistent adipogenic differentiation capacity. This reduces biological variability and justifies the inclusion of a relatively low number of replicates in this pilot study. Also, the use of an immortalized hepatocyte cell line with lower inter-sample variability compared to primary cells allows meaningful interpretation of molecular responses even at lower replicate numbers. In addition, we were unable to analyze the actual PUFA-derived LMs due to small sample volumes, and possible synergistic effects between EV-associated protein/RNA cargo and altered FA profiles could not be investigated.

The EV preparation used in this study likely comprised mixed EV populations and may have included co‑isolated non‑EV material (protein aggregates, lipid droplets, etc.), which could partially contribute to the observed molecular signals and should be considered when interpreting the findings. Although calpeptin reduced EV secretion, the present data do not allow separation of EV‑mediated effects from those of co‑secreted soluble factors, and the hepatocyte responses should therefore be interpreted as secretome‑driven rather than EV‑specific. In addition, a limitation of this study is that CM was collected without a wash step following the calpeptin treatment of adipocytes. As a result, residual calpeptin may have remained in the CM and could have directly affected hepatocytes, representing a potential confounding factor. Another limitation is that the inflammatory phenotype was assessed only at the transcriptional level. While DEGs provide important insight into inflammatory responses, the absence of protein level measurements or downstream signaling readouts decreases the strength of mechanistic conclusions. Consequently, the findings should be interpreted with appropriate caution. Future studies incorporating larger and more heterogeneous sample populations are needed to improve the generalizability of the findings. Calpeptin should also be evaluated in models that better reflect relevant metabolic syndrome phenotypes, such as mice with MASLD, in order to carefully assess the potential pro-inflammatory risks associated with calpeptin treatment. The conclusions drawn from this study are restricted to in vitro cell models, and further validation in vivo using animal experiments is required.

## Conclusion

Although calpeptin has previously demonstrated promising anti-inflammatory, anti-fibrotic, and antiviral potential [8, 41], our results indicate that its metabolic effects require careful and context-specific reassessment. Contrary to our original hypothesis, a new scenario with potentially pro-inflammatory effects emerges. In particular, the impact of calpeptin on lipid metabolism, especially LM pathways and the metabolism of essential PUFAs, should be prioritized in future studies. The observed alterations across multiple pathways suggest that calpeptin may disturb the balance between pro- and anti-inflammatory PUFA-derived metabolites in a complex and potentially unfavorable manner. Moreover, our hepatocyte data emphasize the importance of evaluating tissue-specific and inter-organ consequences of calpain inhibition before advancing toward clinical applications. Thus, the overall therapeutic value of calpeptin remains uncertain, and calpain inhibition may elicit mixed or even adverse effects in the context of metabolic diseases. However, the conclusions of this study are only applicable to in vitro cell models, and the in vivo authenticity of the results requires verification through animal experiments in the future.

## Supplementary Information

Below is the link to the electronic supplementary material.

Supplementary Material 1

Supplementary Material 2

Supplementary Material 3

## Data Availability

All relevant data analyzed during this study are included in this published article and its supplementary information files.

## Abbreviations

ACACA/B: Acetyl-CoA carboxylase alpha/beta
AT: Adipose tissue
AUC: Area under the receiver operating characteristic curve
BSA: Bovine serum albumin
CM: Conditioned medium
CXCL8: C-X-C motif chemokine ligand 8
DEG: Differentially expressed gene
DMA: Plasmalogen alkenyl chain-derived dimethyl acetal derivative
EV: Extracellular vesicle
FA: Fatty acid
GO: Gene Ontology
HFD: High-fat diet
IHH: Immortalized human hepatocyte
IL1B/6: Interleukin 1 beta/6
KEGG: Kyoto Encyclopedia of Genes and Genomes
LM: Lipid mediator
MASLD: Metabolic dysfunction-associated steatotic liver disease
MUFA: Monounsaturated fatty acid
PA: Palmitic acid (16:0)
PBS: Phosphate-buffered saline
PC: Principal component
PCA: Principal component analysis
PL: Phospholipid
PLIN1/4: Perilipin 1/4
PPARG: Peroxisome proliferator-activated receptor gamma
PPI: Protein–protein interaction
PUFA: Polyunsaturated fatty acid
ROC: Receiver operating characteristic
RT-qPCR: Real-time quantitative polymerase chain reaction
SE: Standard error
SFA: Saturated fatty acid
SGBS: Simpson-Golabi-Behmel Syndrome
SREBF1: Sterol regulatory element binding transcription factor 1
STRING: Search Tool for the Retrieval of Interacting Genes/Proteins
TNFA: Tumor necrosis factor alpha
UFA: Unsaturated fatty acid
VIP: Variable importance in projection
