# Supplementary material for: Calpain inhibition by calpeptin modulates adipocyte lipid metabolism and secretome-mediated inflammatory crosstalk with hepatocytes
Source: Inflamm Res. 2026 Jun 4;75(1):132. doi: 10.1007/s00011-026-02280-z (PMC13236743; doi:10.1007/s00011-026-02280-z)
Supplement: Supplementary file 1 — Supplementary Material 1 [file 11_2026_2280_MOESM1_ESM.pdf]

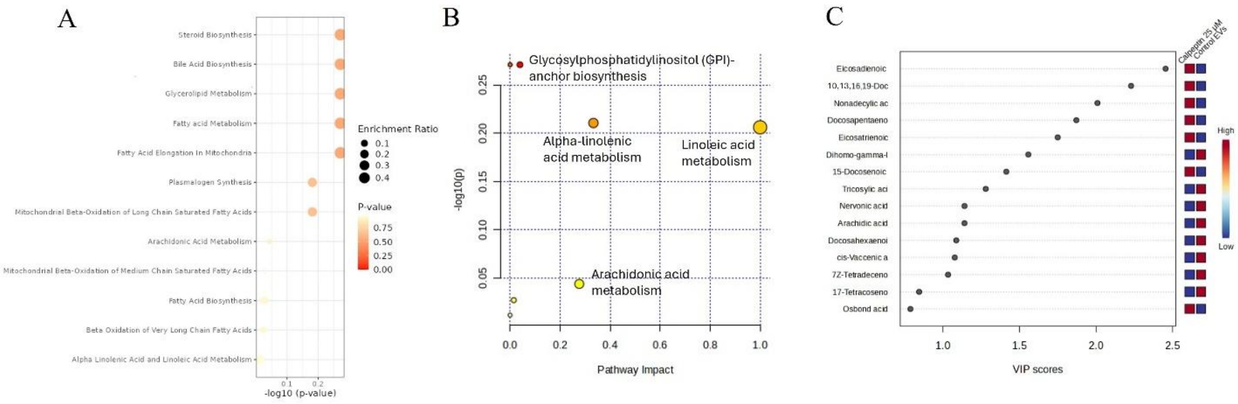

Supplementary Figure S1. Enriched metabolite sets (panel A), summary of metabolic pathway analysis (B), and variable importance in projection (VIP) scores (C) in extracellular vesicles (EVs) derived from Simpson-Golabi-Behmel Syndrome adipocytes treated with 25 μM calpeptin vs. control, analyzed using MetaboAnalyst. Fatty acids are marked by trivial names listed in Supplementary Table S2.
