# Supplementary material for: Calpain inhibition by calpeptin modulates adipocyte lipid metabolism and secretome-mediated inflammatory crosstalk with hepatocytes
Source: Inflamm Res. 2026 Jun 4;75(1):132. doi: 10.1007/s00011-026-02280-z (PMC13236743; doi:10.1007/s00011-026-02280-z)
Supplement: Supplementary file 2 — Supplementary Material 2 [file 11_2026_2280_MOESM2_ESM.pdf]

Supplementary Table S1. Proportions (mol-%) of fatty acids and alkenyl chains, listed in the order of retention times of chromatographic peaks, and their sums and ratios in Simpson-Golabi-Behmel Syndrome adipocytes and their extracellular vesicles (EVs) (mean ± SE).

Fatty acid	Cells, 0 µM	Cells, 25 µM	Cells, 50 µM	EVs, 0 µM	EVs, 25 µM	EVs, 50 µM	*P* Cells[a]	*P* EVs[b]	*P* Cells *vs.* EVs[c]
**N**	4	3	3	4	3	3			
**12:0**	0.216 ± 0.017	0.269 ± 0.038	0.270 ± 0.038	1.958 ± 0.722	2.289 ± 0.950	0.697 ± 0.136	0.407	0.278	**<0.001**
**14:0**	3.084 ± 0.227	3.326 ± 0.188	3.230 ± 0.284	4.154 ± 0.351	4.334 ± 0.179	4.056 ± 0.545	0.689	0.807	**0.006**
**14:1n-9**	0.011 ± 0.002	0.015 ± 0.006	0.014 ± 0.002	0.434 ± 0.151	0.508 ± 0.116	0.323 ± 0.167	0.588	0.624	**<0.001**
**14:1n-7**	0.038 ± 0.004	0.063 ± 0.016	0.047 ± 0.009	0.838 ± 0.230	0.701 ± 0.357	0.177 ± 0.084	0.211	0.120	**0.002**
**14:1n-5**	1.353 ± 0.093	1.639 ± 0.257	1.651 ± 0.287	1.275 ± 0.724	0.902 ± 0.113	0.599 ± 0.236	0.437	0.624	**0.002**
**15:0*i***	0.023 ± 0.005	0.024 ± 0.003	0.043 ± 0.025	1.031 ± 0.461	0.937 ± 0.214	0.533 ± 0.228	0.786	0.624	**<0.001**
**15:0**	0.116 ± 0.018	0.102 ± 0.006	0.106 ± 0.009	0.434 ± 0.112	0.365 ± 0.085	0.223 ± 0.039	0.943	0.269	**<0.001**
**15:1n-6**	0.019 ± 0.006	0.026 ± 0.010	0.029 ± 0.017	1.118 ± 0.559	2.027 ± 1.272	0.259 ± 0.090	0.841	0.366	**<0.001**
**16:0*i***	0.016 ± 0.003	0.026 ± 0.004	0.023 ± 0.002	0.529 ± 0.160	1.235 ± 0.593	0.215 ± 0.045	0.231	0.088	**<0.001**
**DMA 16:0**	0.289 ± 0.036^{B}	0.201 ± 0.028^{A}	0.197 ± 0.017^{A}	0.545 ± 0.145	0.690 ± 0.118	0.607 ± 0.245	**0.038**	0.669	**0.010**
**16:0**	27.043 ± 0.619	27.054 ± 0.835	27.151 ± 1.290	29.461 ± 1.860	25.490 ± 0.999	29.669 ± 3.034	0.905	0.278	0.940
**16:1n-9**	1.262 ± 0.708	0.609 ± 0.129	0.689 ± 0.169	0.319 ± 0.035	0.282 ± 0.015	0.292 ± 0.068	0.905	0.694	**<0.001**
**16:1n-7**	31.557 ± 1.547	35.909 ± 2.720	35.857 ± 1.833	1.558 ± 0.184	1.416 ± 0.101	1.362 ± 0.035	0.215	0.750	**<0.001**
**16:1n-5**	0.050 ± 0.007	0.036 ± 0.002	0.038 ± 0.004	0.980 ± 0.367	0.650 ± 0.089	0.866 ± 0.349	0.137	0.807	**<0.001**
**17:0*i***	0.042 ± 0.003	0.056 ± 0.014	0.064 ± 0.012	0.386 ± 0.091	0.834 ± 0.162	0.564 ± 0.318	0.150	0.155	**<0.001**
**17:0*ai***	0.044 ± 0.006	0.037 ± 0.005	0.038 ± 0.008	0.794 ± 0.204	0.852 ± 0.272	0.471 ± 0.047	0.689	0.366	**<0.001**
**17:0**	0.078 ± 0.014	0.064 ± 0.019	0.051 ± 0.006	1.779 ± 1.120	1.985 ± 0.793	0.583 ± 0.096	0.317	0.624	**<0.001**
**17:1n-8**	0.232 ± 0.032	0.194 ± 0.012	0.210 ± 0.015	1.166 ± 0.711	0.993 ± 0.644	0.213 ± 0.013	0.841	0.624	0.173
**18:0*i***	0.028 ± 0.007	0.032 ± 0.008	0.036 ± 0.010	1.494 ± 0.589	1.485 ± 0.729	0.683 ± 0.212	0.237	0.657	**<0.001**
**DMA 18:0**	0.119 ± 0.025	0.063 ± 0.014	0.069 ± 0.004	0.479 ± 0.236	0.651 ± 0.208	0.210 ± 0.057	0.064	0.268	**<0.001**
**DMA 18:1n-9**	0.064 ± 0.010	0.057 ± 0.025	0.044 ± 0.003	1.410 ± 0.779	1.904 ± 0.709	1.276 ± 0.924	0.490	0.778	**<0.001**
**DMA 18:1n-7**	0.119 ± 0.013	0.090 ± 0.018	0.092 ± 0.004	1.265 ± 0.689	1.946 ± 0.408	1.144 ± 0.536	0.237	0.520	**<0.001**
**18:0**	1.710 ± 0.195	1.460 ± 0.382	1.324 ± 0.221	18.811 ± 0.700	18.083 ± 2.718	18.611 ± 1.748	0.490	0.694	**<0.001**
**18:1n-9**	18.928 ± 1.318	17.832 ± 0.935	17.785 ± 1.054	14.304 ± 5.554	12.475 ± 6.525	21.536 ± 3.076	0.786	0.388	0.940
**18:1n-7**	9.933 ± 0.811	8.109 ± 0.902	8.490 ± 0.404	1.295 ± 0.371	1.218 ± 0.569	1.969 ± 0.385	0.231	0.386	**<0.001**
**18:1n-5**	0.688 ± 0.028^{B}	0.573 ± 0.019^{AB}	0.552 ± 0.032^{A}	0.523 ± 0.178	0.925 ± 0.429	1.182 ± 0.548	**0.035**	0.669	0.545
**18:2n-6**	0.148 ± 0.025	0.138 ± 0.031	0.105 ± 0.008	1.381 ± 0.133	1.218 ± 0.110	1.379 ± 0.226	0.317	0.648	**<0.001**
**18:3n-6**	0.015 ± 0.006	0.013 ± 0.003	0.014 ± 0.002	0.418 ± 0.109	0.521 ± 0.157	0.395 ± 0.260	0.978	0.657	**<0.001**
**19:0**	0.018 ± 0.010	0.028 ± 0.011	0.012 ± 0.003	0.223 ± 0.068	0.470 ± 0.128	0.490 ± 0.140	0.943	0.231	**<0.001**
**19:1n1:0**	0.030 ± 0.008	0.023 ± 0.009	0.012 ± 0.001	0.900 ± 0.317	0.838 ± 0.133	0.413 ± 0.126	0.283	0.269	**<0.001**
**19:1n-8**	0.076 ± 0.025	0.038 ± 0.009	0.028 ± 0.004	0.466 ± 0.315	0.443 ± 0.279	0.642 ± 0.542	0.112	0.900	**<0.001**
**18:3n-3**	0.017 ± 0.003	0.011 ± 0.004	0.008 ± 0.002	1.107 ± 0.498	1.193 ± 0.302	0.806 ± 0.423	0.082	0.864	**<0.001**
**20:0**	0.045 ± 0.009	0.031 ± 0.006	0.033 ± 0.005	0.347 ± 0.079^{B}	0.240 ± 0.015^{AB}	0.154 ± 0.055^{A}	0.567	**0.030**	**<0.001**
**20:1n-9**	0.267 ± 0.048	0.200 ± 0.037	0.161 ± 0.005	0.228 ± 0.049	0.222 ± 0.027	0.251 ± 0.034	0.150	0.900	0.496
**20:1n-7**	0.206 ± 0.024	0.167 ± 0.031	0.144 ± 0.003	0.333 ± 0.109	0.352 ± 0.096	0.334 ± 0.191	0.150	0.900	0.082
**20:2n-9**	0.098 ± 0.029	0.077 ± 0.025	0.049 ± 0.006	0.717 ± 0.514	1.191 ± 0.625	1.193 ± 0.864	0.490	0.423	**0.016**
**20:2n-6**	0.018 ± 0.007	0.012 ± 0.005	0.015 ± 0.006	0.316 ± 0.152	0.552 ± 0.107	0.639 ± 0.373	0.845	0.300	**<0.001**
**20:3n-9**	0.363 ± 0.025	0.291 ± 0.034	0.297 ± 0.012	0.115 ± 0.056	0.103 ± 0.013	0.107 ± 0.040	0.215	0.968	**<0.001**
**20:3n-6**	0.048 ± 0.018	0.028 ± 0.011	0.028 ± 0.002	0.470 ± 0.165	0.306 ± 0.178	0.275 ± 0.182	0.705	0.648	**<0.001**
**20:4n-6**	0.411 ± 0.111^{B}	0.227 ± 0.026^{A}	0.241 ± 0.011^{A}	0.576 ± 0.168	0.653 ± 0.226	0.255 ± 0.117	**0.038**	0.269	0.496
**20:3n-3**	0.005 ± 0.001	0.007 ± 0.001	0.005 ± 0.001	0.124 ± 0.077	0.133 ± 0.049	0.049 ± 0.020	0.328	0.401	**<0.001**
**20:4n-3**	0.012 ± 0.003	0.008 ± 0.003	0.008 ± 0.001	0.304 ± 0.068	0.687 ± 0.556	0.693 ± 0.556	0.689	0.913	**<0.001**
**20:5n-3**	0.068 ± 0.012	0.020 ± 0.008	0.044 ± 0.014	0.313 ± 0.117	0.283 ± 0.010	0.272 ± 0.137	0.078	0.968	**0.001**
**22:0**	0.045 ± 0.006	0.030 ± 0.010	0.035 ± 0.007	0.263 ± 0.069	0.350 ± 0.022	0.309 ± 0.100	0.352	0.807	**<0.001**
**22:1n-9**	0.055 ± 0.010	0.020 ± 0.005	0.030 ± 0.007	0.139 ± 0.030	0.115 ± 0.018	0.089 ± 0.039	0.066	0.520	**0.003**
**22:1n-7**	0.012 ± 0.001	0.011 ± 0.002	0.010 ± 0.003	0.052 ± 0.016	0.091 ± 0.026	0.060 ± 0.023	0.689	0.648	**<0.001**
**22:2n-9**	0.014 ± 0.004	0.013 ± 0.005	0.009 ± 0.003	0.191 ± 0.090	0.183 ± 0.079	0.136 ± 0.077	0.445	0.788	**<0.001**
**22:3n-9**	0.165 ± 0.035^{B}	0.071 ± 0.018^{AB}	0.035 ± 0.015^{A}	0.387 ± 0.143	0.374 ± 0.185	0.612 ± 0.249	**0.046**	0.719	**0.004**
**23:0**	0.044 ± 0.022	0.193 ± 0.086	0.122 ± 0.020	0.149 ± 0.032	0.098 ± 0.021	0.059 ± 0.019	0.102	0.208	0.705
**22:4n-6**	0.179 ± 0.048	0.088 ± 0.015	0.101 ± 0.010	0.067 ± 0.012	0.090 ± 0.029	0.110 ± 0.072	0.062	0.864	0.070
**22:5n-6**	0.045 ± 0.007^{B}	0.016 ± 0.004^{AB}	0.016 ± 0.002^{A}	0.122 ± 0.024	0.208 ± 0.098	0.206 ± 0.112	**0.035**	0.694	**0.002**
**22:4n-3**	0.007 ± 0.002	0.004 ± 0.001	0.007 ± 0.003	0.056 ± 0.022	0.119 ± 0.026	0.130 ± 0.058	0.588	0.439	**<0.001**
**22:5n-3**	0.181 ± 0.029	0.120 ± 0.020	0.104 ± 0.007	0.403 ± 0.143	0.775 ± 0.178	0.663 ± 0.243	0.082	0.167	**<0.001**
**24:0**	0.062 ± 0.003	0.060 ± 0.020	0.040 ± 0.007	0.189 ± 0.045	0.222 ± 0.050	0.154 ± 0.056	0.186	0.657	**0.001**
**22:6n-3**	0.215 ± 0.056	0.135 ± 0.024	0.136 ± 0.007	1.043 ± 0.267	2.607 ± 2.310	0.660 ± 0.482	0.317	0.750	**0.019**
**24:1n-9**	0.064 ± 0.009	0.043 ± 0.018	0.044 ± 0.010	0.177 ± 0.046	0.124 ± 0.033	0.105 ± 0.046	0.317	0.223	**0.001**
**24:1n-7**	0.007 ± 0.001	0.009 ± 0.003	0.007 ± 0.002	0.082 ± 0.029	0.063 ± 0.021	0.041 ± 0.024	0.727	0.379	**<0.001**
**∑:SFA**	32.613 ± 0.335	32.792 ± 0.959	32.579 ± 1.147	62.003 ± 2.611	59.267 ± 5.590	57.469 ± 3.433	0.689	0.807	**<0.001**
**∑:MUFA**	64.788 ± 0.326	65.515 ± 1.227	65.797 ± 1.139	26.187 ± 3.975	24.345 ± 5.207	30.714 ± 1.722	0.554	0.520	**<0.001**
**∑:PUFA**	2.009 ± 0.362^{B}	1.281 ± 0.200^{AB}	1.222 ± 0.038^{A}	8.111 ± 1.612	11.198 ± 3.281	8.580 ± 3.221	**0.035**	0.788	**<0.001**
**∑:n-6 PUFA**	0.864 ± 0.201^{B}	0.523 ± 0.076^{AB}	0.520 ± 0.016^{A}	3.351 ± 0.449	3.550 ± 0.431	3.259 ± 0.800	**0.035**	0.864	**<0.001**
**∑:n-3 PUFA**	0.505 ± 0.096	0.306 ± 0.052	0.311 ± 0.015	3.350 ± 0.580	5.797 ± 2.225	3.272 ± 1.313	0.064	0.900	**<0.001**
**UFA/SFA**	2.049 ± 0.030	2.042 ± 0.094	2.065 ± 0.113	0.564 ± 0.088	0.629 ± 0.155	0.693 ± 0.076	0.689	0.669	**<0.001**
**n-3/n-6 PUFA**	0.599 ± 0.038	0.582 ± 0.033	0.598 ± 0.015	0.985 ± 0.104	1.563 ± 0.431	0.937 ± 0.312	0.841	0.499	**0.002**
**∑:DMA**	0.591 ± 0.077	0.412 ± 0.081	0.403 ± 0.026	3.699 ± 1.791	5.191 ± 0.987	3.237 ± 1.245	0.082	0.499	**<0.001**
**Prod/prec n-6**	2.988 ± 0.358	1.971 ± 0.294	2.573 ± 0.090	0.837 ± 0.304	0.840 ± 0.320	0.490 ± 0.337	0.132	0.624	**<0.001**
**Prod/prec n-3**	27.995 ± 4.175	28.838 ± 8.217	40.750 ± 11.743	3.046 ± 1.133	2.953 ± 1.530	2.130 ± 0.144	0.585	0.750	**<0.001**
**Δ6-DI n-6**	0.123 ± 0.058	0.115 ± 0.055	0.133 ± 0.031	0.320 ± 0.083	0.456 ± 0.156	0.376 ± 0.286	0.786	0.657	**0.034**
**Δ5-DI n-6**	10.272 ± 2.177	10.009 ± 2.992	8.654 ± 0.550	1.385 ± 0.213	3.135 ± 1.563	1.232 ± 0.362	0.978	0.269	**<0.001**
**Δ5-DI n-3**	8.199 ± 3.738	2.960 ± 1.005	5.422 ± 1.765	1.094 ± 0.319	1.682 ± 0.939	0.995 ± 0.539	0.317	0.864	**0.001**

i = *iso*-methyl-branch, *ai* = *anteiso*-methyl-branch, DMA = dimethyl acetal (derivative of alkenyl chain), SFA = saturated fatty acid, MUFA = monounsaturated fatty acid, PUFA = polyunsaturated fatty acid, UFA = unsaturated fatty acid (MUFA + PUFA), Prod/Prec n-6 = product/precursor n-6 PUFAs (20:3n-6 + 20:4n-6)/18:2n-6, Prod/prec n-3 = product/precursor n-3 PUFAs (20:5n-3 + 22:5n-3 + 22:6n-3)/18:3n-3, Δ6-DI n-6 = Δ6-desaturation index n-6 PUFAs (18:3n-6/18:2n-6) Δ5-DI n-6 = Δ5-desaturation index n-6 PUFAs (20:4n-6/20:3n-6), Δ5-DI n-3 = Δ5-desaturation index n-3 PUFAs (20:5n-3/20:4n-3) [a]the effect of group within 0, 25, and 50 µM cells, [b]the effect of group within 0, 25, and 50 µM EVs (Kruskal–Wallis test), [c]the effect of sample type between pooled 0, 25, and 50 µM cells and pooled 0, 25, and 50 µM EVs (Mann–Whitney U test), bold *p*-values and dissimilar superscript capital letters indicate significant differences between groups within cells or EVs, or between cells and EVs.
