# Supplementary material for: Calpain inhibition by calpeptin modulates adipocyte lipid metabolism and secretome-mediated inflammatory crosstalk with hepatocytes
Source: Inflamm Res. 2026 Jun 4;75(1):132. doi: 10.1007/s00011-026-02280-z (PMC13236743; doi:10.1007/s00011-026-02280-z)
Supplement: Supplementary file 3 — Supplementary Material 3 [file 11_2026_2280_MOESM3_ESM.pdf]

Supplementary Table S2. Trivial names of fatty acids (FAs) used in Figures 3–5 and in Supplementary Fig. S1 based on the MetaboAnalyst platform.

FA shorthand notation	Trivial FA name
12:0	Lauric acid
14:0	Myristic acid
14:1n-9	5Z-Tetradecenoic acid
14:1n-7	7Z-Tetradecenoic acid
14:1n-5	Myristoleic acid
15:0	Pentadecylic acid
16:0	Palmitic acid
16:1n-9	Hypogeic acid
16:1n-7	Palmitoleic acid
17:0	Margaric acid
17:1n-8	9Z-Heptadecenoic acid
18:0	Stearic acid
18:1n-9	Oleic acid
18:1n-7	cis-Vaccenic acid
18:2n-6	Linoleic acid
18:3n-6	gamma-Linolenic acid
19:0	Nonadecylic acid
19:1n-10	cis-9-Nonadecenoic acid
18:3n-3	alpha-Linolenic acid
20:0	Arachidic acid
20:1n-9	11-Eicosenoic acid
20:1n-7	Paullinate
20:2n-6	Eicosadienoic acid
20:3n-9	Mead acid
20:3n-6	Dihomo-gamma-linolenic acid
20:4n-6	Arachidonic acid
20:3n-3	Eicosatrienoic acid
20:4n-3	8,11,14,17-Eicosatetraenoic acid
20:5n-3	Eicosapentaenoic acid
22:0	Behenic acid
22:1n-9	Erucic acid
22:1n-7	15-Docosenoic acid
23:0	Tricosylic acid
22:4n-6	Adrenic acid
22:5n-6	Osbond acid
22:4n-3	10,13,16,19-Docosatetraenoic acid
22:5n-3	Clupanodonic acid
24:0	Lignoceric acid
22:6n-3	Docosahexaenoic acid
24:1n-9	Nervonic acid
24:1n-7	17(Z)-Tetracosenoic acid
